# Microneurotrophin BNN27 Exerts Significant Anti-Inflammatory Effects on Murine T-Lymphocytes Following CFA-Induced Inflammatory Pain

**DOI:** 10.3390/ijms26125498

**Published:** 2025-06-08

**Authors:** Smaragda Poulaki, Aikaterini Kalantidou, Ioanna Lapi, Achille Gravanis, Maria Venihaki

**Affiliations:** 1 Department of Clinical Chemistry, Medical School, University of Crete, 71500 Heraklion, Crete, Greece; s.poulaki@med.uoc.gr (S.P.); medp2011987@med.uoc.gr (A.K.); medp2011988@med.uoc.gr (I.L.); 2 Department of Pediatrics, School of Medicine, University of Crete, 71500 Heraklion, Crete, Greece; 3 Institute of Molecular Biology & Biotechnology, Foundation of Research & Technology-Hellas, 70013 Heraklion, Crete, Greece; gravanis@med.uoc.gr; 4 Department of Pharmacology, Medical School, University of Crete, 71500 Heraklion, Crete, Greece

**Keywords:** microneurotrophin, BNN27, CFA, T-lymphocytes, interleukins, analgesia

## Abstract

During tissue injury or infection, leukocytes are activated to produce proinflammatory mediators, which trigger the immune system to produce anti-inflammatory and analgesic molecules. Our previous studies provide evidence that synthetic microneurotrophins, like BNN27, exert significant analgesic and anti-inflammatory effects during Complete Freund’s Adjuvant (CFA)-induced inflammation and pain. Thus, the aim of the present study was to examine if the effect of BNN27 on inflammatory pain is mediated at least in part by activation of T-lymphocytes. For this purpose, six hours following the injection of CFA, spleens were harvested in PBS and lymphocytes were collected and placed in medium containing concanavalin-A and IL-2 to prompt T-lymphocyte proliferation and differentiation. Cells were then treated with BNN27 at different concentrations and the media and cells were collected for ELISA and PCR assays. The proliferation rate of T-cells was also examined using the MTT assay. Our results showed that BNN27 significantly increased the proliferation of T-lymphocytes. In addition, BNN27 significantly decreased IL-6 and TNF-α protein levels, while it increased the mRNA expression of μ-opioid receptor and opioid peptides PENK and POMC at different time points. Our data demonstrate considerable anti-inflammatory and analgesic effects of BNN27, making it a promising molecule for inflammation and pain management.

## 1. Introduction

Lymphocytes are key cells of the immune system that mediate adaptive immunity mechanisms. They are divided into T lymphocytes which are responsible for cell mediated adaptive immunity and B lymphocytes that contribute to antibody formation in response to antigens [1,2]. Both T and B lymphocytes originate from a common lymphoid progenitor in the bone marrow; however, T cells migrate and mature in thymus, where they differentiate into three major subtypes: T helper cell (CD4), cytotoxic T cells (CD8) and regulatory T cells (CD4) [3,4].

Upon antigen presence, T helper cells secrete cytokines that attract cytotoxic T cells and macrophages at the infected site, which attack cells infected by bacteria or viruses or tumour cells [5,6]. Following the elimination of the threat, regulatory T cells are activated to suppress the immune response to prevent cytotoxic T cells from attacking healthy cells [7]. During this process memory T cells are also formed to ensure a faster immune response upon infection by the same antigen [8].

Various inflammatory situations are characterized by the recruitment of T cells in order to counteract inflammation [9]; however, multiple pathogenic conditions are characterized by the accumulation of T cell subtypes which seem to further enhance inflammation. When the immune system is unable to eliminate pathogens, immune cells such as neutrophils and T cells continuously present within inflamed tissues leading to persistent chronic inflammation, autoimmune diseases, or tumourigenesis [10,11,12,13].

Neurosteroids have been shown to either potentiate or to inhibit different lymphocyte functions such as proliferation [14], cytokine secretion [15,16] and differentiation [16,17]. Based on the above, the aim of the present study was to evaluate the effect of the synthetic spiro-epoxy analogue of Dehydroepiandrosterone (DHEA), BNN27 on T lymphocytes derived from mouse spleens which were activated by an intraplantar injection of Complete Freund’s Adjuvant (CFA) into the mouse hind paw which triggers local and systemic inflammatory response [18].

## 2. Results

### 2.1. BNN27 Affected T Lymphocytes Proliferation in a Concentration-Dependent Manner

**Figure 1:**
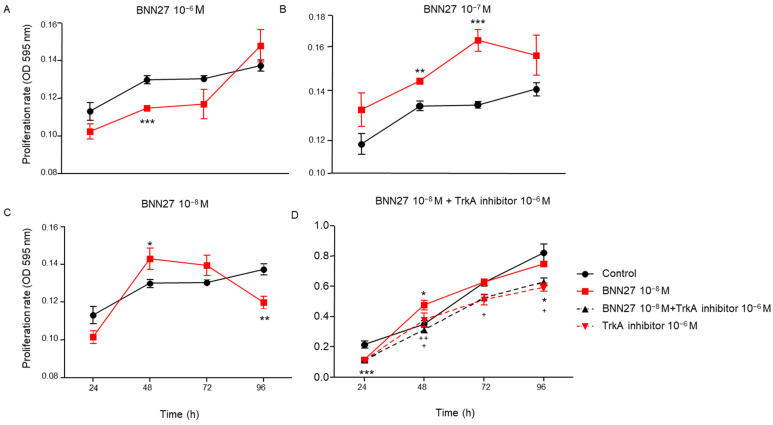
T lymphocyte proliferation rate following treatment with different concentrations of BNN27 and/or TrkA inhibitor. T cell proliferation rate was decreased at 48 h with 10^−6^ M BNN27 (**A**), while 10^−7^ M BNN27 led to increased proliferation rate at both 48 and 72 h (**B**). Treatment with 10^−8^ M BNN27 resulted in elevated proliferation rate at 48 h, followed by a decline at 96 h (**C**). When BNN27 10^−8^ M was combined with a TrkA inhibitor, T cell proliferation rate was reduced at 48, 72, and 96 hours compared to treatment with 10^−8^ M BNN27 alone (**D**). (*) Represents comparison between the control group and BNN27-treated group, (⁺) represents comparison between BNN27-treated group and BNN27 + TrkA inhibitor-treated group. In all panels: n = 6. * *p* < 0.05, ** *p* < 0.01, *** *p* < 0.001. ⁺ *p* < 0.05, ⁺⁺ *p* < 0.01. Statistical analysis was performed using 2-way ANOVA followed by Bonferroni post-hoc test. Data are expressed as mean ± SEM.

To assess the effect of BNN27 on T lymphocytes proliferation rate, the compound was added in T lymphocytes culture media in different concentrations (10^−6^ M, 10^−7^ M and 10^−8^ M). Our results demonstrate that BNN27 at 10^−6^ M significantly decreased the proliferation rate of T lymphocytes 48 h following the addition in the culture media (Figure 1A), while BNN27 at 10^−7^ M significantly increased T lymphocytes proliferation 48 and 72 h post-treatment (Figure 1B). At the concentration of 10^−8^ M, we observed a decreased proliferation rate of T lymphocytes at 96 h (Figure 1C). To verify that the effect of BNN27 was not due to cell death, we performed cell viability assays using Trypan blue. Our results showed that there was no difference in the viability ([(total cells − dead cells)/total cells] × 100) between vehicle- and BNN27-treated cells at the concentration of 10^−8^ M at 96 h (viability: vehicle-treated cells: 40 ± 4; BNN27-treated-cells: 34 ± 2, n = 5 wells/treatment/experiment).

Treatment of cells with BNN27 (10^−8^ M) and the specific TrkA inhibitor (10^−6^ M) simultaneously resulted in a decreased proliferation rate of T lymphocytes compared to BNN27 10^−8^ M treated group at 48, 72 and 96 h (Figure 1D).

### 2.2. Effect of BNN27 on T Lymphocyte Secreted Cytokines

**Figure 2:**
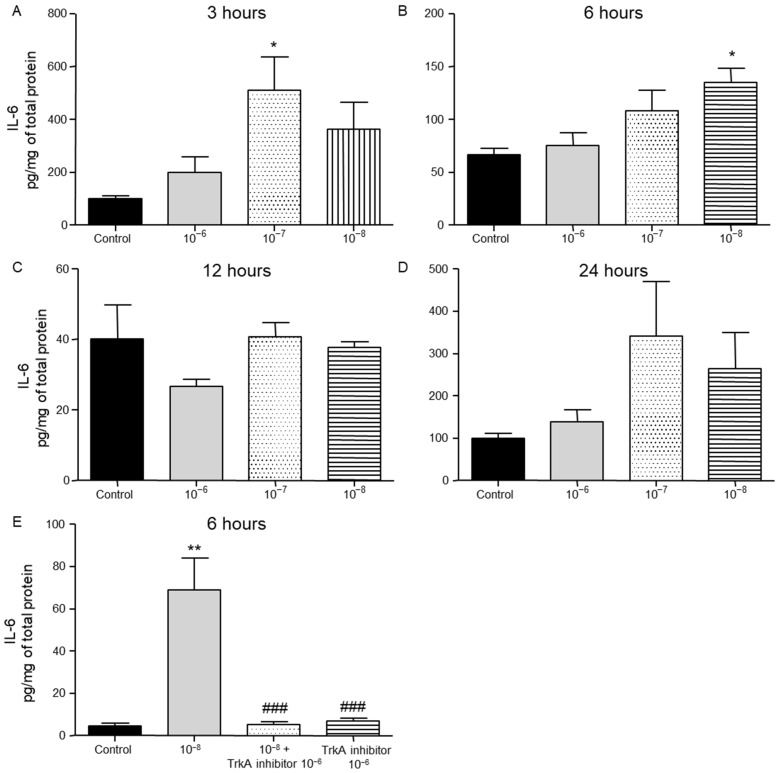
Effect of BNN27 on IL-6 protein levels. Administration of BNN27 10^−7^ M increased IL-6 protein levels at 3 h (**A**), while BNN27 at 10^−8^ M increased IL-6 at 6 h (**B**). At 12 and 24 h following treatment of T lymphocytes with BNN27 no changes were observed at any of the concentrations tested (**C**,**D**). Addition of TrkA inhibitor to the culture resulted in reduced IL-6 protein levels at 6 h (**E**). (*) Represents comparison between control group and BNN27-treated group, (###) represents comparison between BNN27-treated group and TrkA inhibitor, and BNN27 + TrkA inhibitor-treated groups. (**A**) Control group n = 6, BNN27 10^−6^ M n = 6, BNN27 10^−7^ M n = 6, BNN27 10^−8^ M n = 6; (**B**) in all groups n = 6; (**C**) in all groups n = 3; (**D**) in all groups n = 6; (**E**) control n = 3, BNN27 10^−8^ M n = 4, BNN27 + TrkA inhibitor n = 4, TrkA inhibitor n = 3. In all panels: * *p* < 0.05, ** *p* < 0.01, ^(###)^ *p* < 0.001. Statistical analysis was performed using 1-way ANOVA and Newman–Keuls post-hoc test. Data are expressed as mean ± SEM.

We then evaluated the effect of different concentrations of BNN27 on the release of cytokines from T lymphocytes. IL-6 protein levels were elevated following treatment with 10^−7^ M BNN27 at 3 h (Figure 2A), while at 6 h IL-6 protein levels were significantly elevated following treatment of T lymphocytes with 10^−8^ M BNN27 (Figure 2B). Twelve and twenty-four hours after treatment with BNN27 at any concentration no significant changes in IL-6 protein levels were observed (Figure 2C,D). Treatment with BNN27 and TrkA or TrkA inhibitor alone significantly reduced IL-6 protein levels compared to BNN27 10^−8^ M treated group at 6 h (Figure 2E).

**Figure 3:**
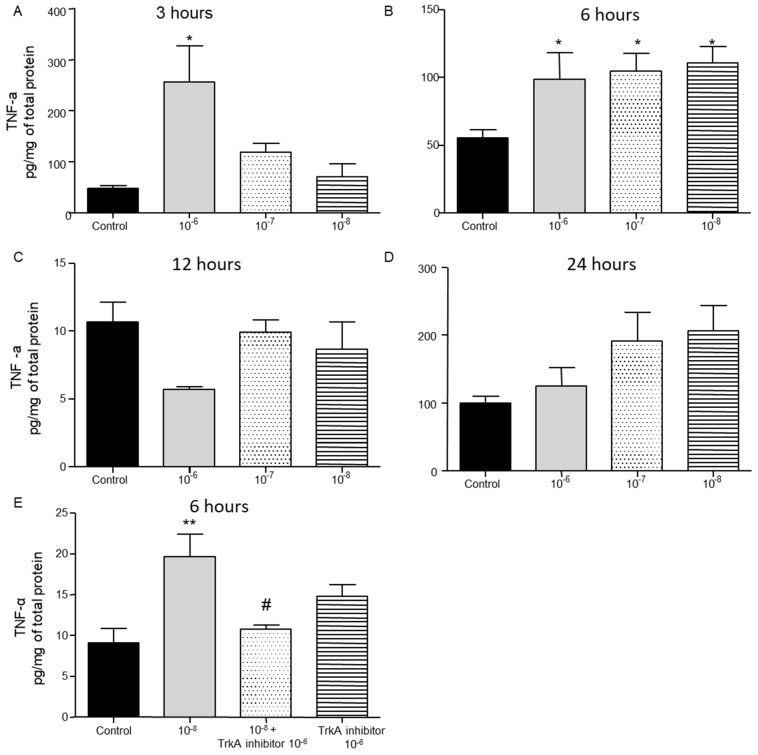
Effect of BNN27 on TNF-α protein levels. T lymphocytes treated with BNN27 10^−6^ M exhibited elevated TNF-α levels at 3 h (**A**). All doses of BNN27 increased TNF-α protein levels at 6 h (**B**). At 12 and 24 h BNN27 did not affect TNF-α protein levels (**C**,**D**). Inhibition of TrkA resulted in decreased TNF-α protein levels at 6 h (**E**). (*) Represents comparison between control and BNN27-treated group, (#) represents comparison between BNN27-treated group and TrkA inhibitor, and BNN27 + TrkA inhibitor-treated groups. (**A**) In all groups n = 3; (**B**) in all groups n = 6; (**C**) in all groups n = 3; (**D**) in all groups n = 6; (**E**) in all groups n = 4. In all panels: * *p* < 0.05, ** *p* < 0.01, ^(#)^ *p* < 0.05. Statistical analysis was performed using 1-way ANOVA and Newman–Keuls post-hoc test. Data are expressed as mean ± SEM.

Administration of BNN27 10^−6^ M resulted in increased TNF-α protein levels at 3 h following its addition to the culture (Figure 3A), while at 6 h TNF-α protein levels were found significantly elevated at all concentrations tested (Figure 3B). No changes in TNF-α levels were detected 12 and 24 h after the addition of BNN27 to the T lymphocyte culture (Figure 3C,D). Pharmacological blockade of the TrkA receptor resulted in significantly reduced protein levels of TNF-α compared to BNN27 10^−8^ M treated at 6 h (Figure 3E).

**Figure 4:**
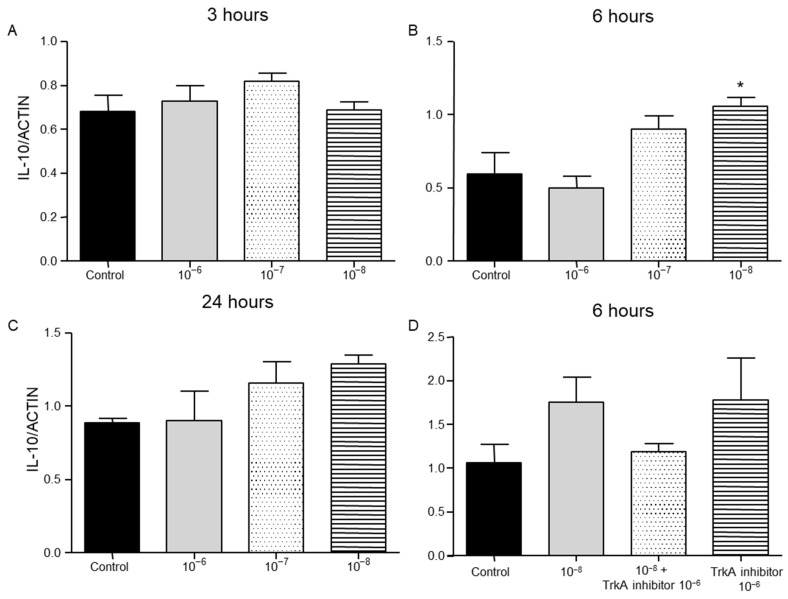
Effect of BNN27 on IL-10 expression. T-lymphocytes treated with BNN27 10^−8^ M showed significantly elevated IL-10 mRNA levels, only at 6 h post treatment (**B**). (*) Represents comparison between control and BNN27-treated group. (**A**) In all groups n = 3; (**B**) in all groups n = 3; (**C**) in all groups n = 3; (**D**) control n = 4, BNN27 10^−8^ M n = 4, BNN27 + TrkA inhibitor n = 3, TrkA inhibitor n = 4. In all panels * *p* < 0.05. Statistical analysis was performed with 1-way ANOVA and Newman–Keuls post-hoc test. Data are expressed as mean ± SEM.

IL-10 protein levels were not detectable in T lymphocytes culture. However, IL-10 mRNA was detected 3, 6 and 24 h after the treatment of T lymphocytes with BNN27. Treatment with BNN27 10^−8^ M increased IL-10 expression 6 h after its administration in the culture (Figure 4B), while at other concentrations and intervals studied, no apparent changes were observed on IL-10 expression levels (Figure 4A,C,D).

### 2.3. Effect of BNN27 on NGF and Its Receptor TrkA Synthesis by T Lymphocytes

**Figure 5:**
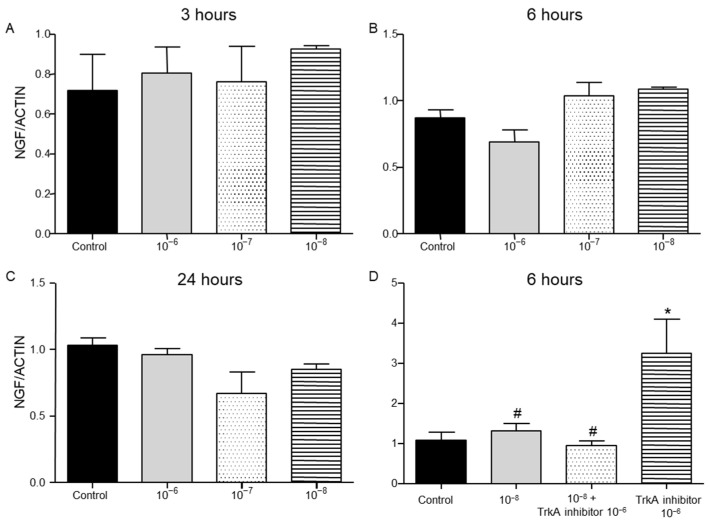
Effect of BNN27 and TrkA inhibitor on NGF. BNN27 did not affect NGF expression in T lymphocytes (**A**–**C**). Inhibition of TrkA resulted in increased NGF mRNA levels compared to control at 6 h, whereas co-administration of BNN27 with TrkA inhibitor decreased NGF mRNA levels (**D**). (*) Represents comparison between control group and BNN27 + TrkA inhibitor-treated group, (#) represents comparison between BNN27-treated group and TrkA inhibitor, and BNN27 + TrkA inhibitor-treated groups. (**A**) In all groups n = 3; (**B**) in all groups n = 3; (**C**) in all groups n = 3; (**D**) control n = 4, BNN27 10^−8^ M n = 4, BNN27 + TrkA inhibitor n = 3, TrkA inhibitor n = 4. In all panels * *p* < 0.05. Statistical analysis was performed using 1-way ANOVA and Newman–Keuls post-hoc test. Data are expressed as mean ± SEM. In all panels: * *p* < 0.05, ^(#)^ *p* < 0.05. Statistical analysis was performed using 1-way ANOVA and Newman–Keuls post-hoc test. Data are expressed as mean ± SEM.

Addition of BNN27 to T lymphocyte culture did not significantly affect NGF mRNA expression at any time point or concentrations examined (Figure 5A–C). Co-administration of BNN27 with TrkA receptor inhibitor significantly decreased NGF synthesis by T lymphocytes, while the opposite effect was observed following treatment only with TrkA inhibitor (Figure 5D).

**Figure 6:**
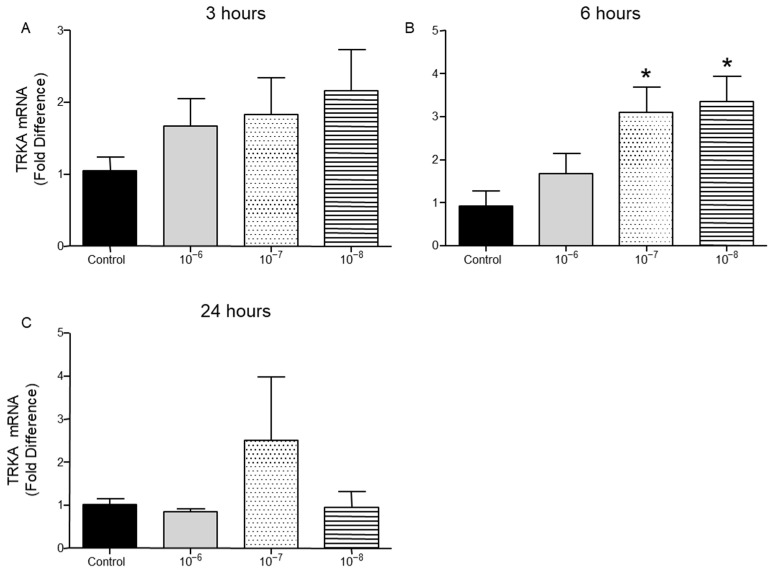
Effect of BNN27 on TrkA receptor mRNA. Elevated TrkA mRNA levels were detected in T-lymphocytes after administration of BNN27 at 10^−7^ M and 10^−8^ M at 6 h (**B**). (**A**) In all groups n = 3; (**B**) in all groups n = 3; (**C**) in all groups n = 3. (*) Indicates statistically significant difference between BNN27-treated group or BNN27 + TrkA inhibitor and the Control group. In all panels: * *p* < 0.05. Statistical analysis was performed using one-way ANOVA and Newman–Keuls post-hoc test. Data are expressed as mean ± SEM.

TrkA receptor mRNA levels were not affected by treatment of T lymphocytes with BNN27 at 3 h at any concentration examined (Figure 6A). At 6 h post treatment with BNN27 at 10^−7^ M and 10^−8^ M, TrkA mRNA levels were significantly increased (Figure 6B). At 24 h, no significant change in TrkA mRNA levels was observed (Figure 6C).

### 2.4. Effect of BNN27 on Opioids Synthesis by T Lymphocytes

**Figure 7:**
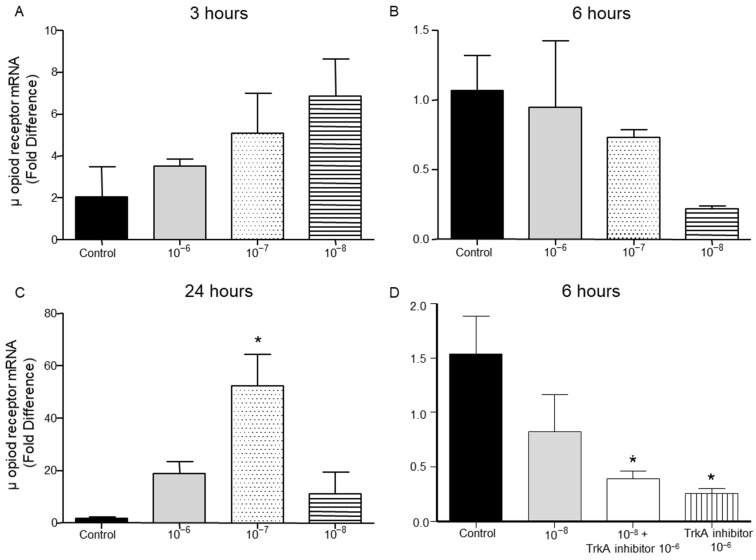
Effect of BNN27 on μ opioid receptor mRNA. Elevated μ opioid receptor mRNA levels were detected in T-lymphocytes following treatment with BNN27 10^−7^ at 24 h (**C**). Addition of TrkA inhibitor decreased μ opioid receptor expression (**D**). (**A**) In all groups n = 3; (**B**) in all groups n = 3; (**C**) in all groups n = 3; (**D**) in all groups n = 3. (*) Indicates statistically significant difference between BNN27-treated group or BNN27 + TrkA inhibitor and the control group. In all panels: * *p* < 0.05. Statistical analysis was performed using one-way ANOVA and Newman–Keuls post-hoc test. Data are expressed as mean ± SEM.

It is well established that opioid peptides are also expressed in the periphery by immune cells such as T lymphocytes [19]. In the present study, treatment of T lymphocytes with different concentrations of BNN27 did not affect μ opioid receptor mRNA levels at 3 and 6 h (Figure 7A,B). However, at 24 h the addition of BNN27 at 10^−7^ M to the culture media significantly increased μ opioid receptor mRNA levels (Figure 7C). Pharmacological blockade of the NGF receptor, TrkA, significantly reduced μ opioid receptor mRNA levels at 6 h (Figure 7D).

**Figure 8:**
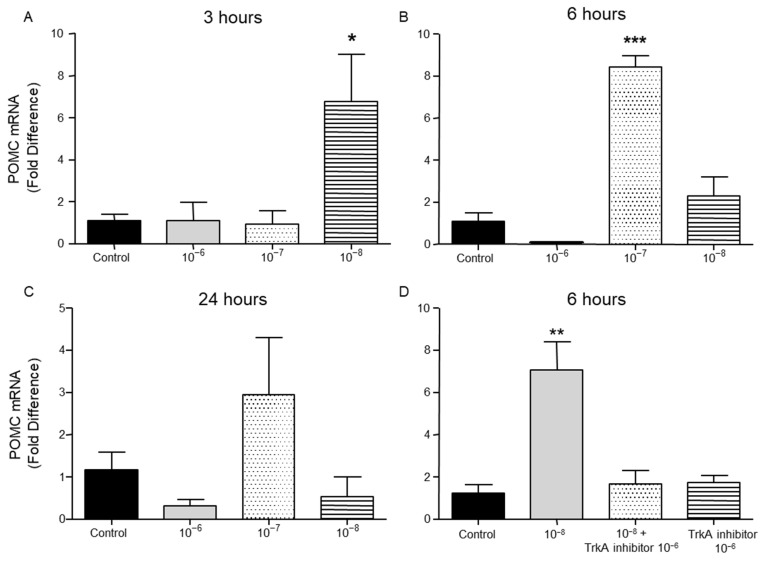
Effect of BNN27 on POMC mRNA levels. Elevated POMC mRNA levels were detected after administration of BNN27 10^−8^ M at 3 h (**A**). Six (6) hours after administration of BNN27 at 10^−7^ M POMC synthesis was increased by T lymphocytes (**B**) while at 24 h POMC mRNA levels were not affected by BNN27 administration (**C**). Inhibition of TrkA resulted in reduced POMC mRNA levels at 6 h (**D**). (*) Represents comparison between control group and BNN27-treated group. (**A**) In all groups n = 3; (**B**) in all groups n = 3; (**C**) in all groups n = 3; (**D**) control group n = 4, BNN27 10^−8^ M n = 4, BNN27 + TrkA inhibitor n = 3, TrkA inhibitor n = 4. In all panels: * *p* < 0.05, ** *p* < 0.01, *** *p* < 0.001. Statistical analysis was performed using 1-way ANOVA and Newman–Keuls post-hoc test. Data are expressed as mean ± SEM.

**Figure 9:**
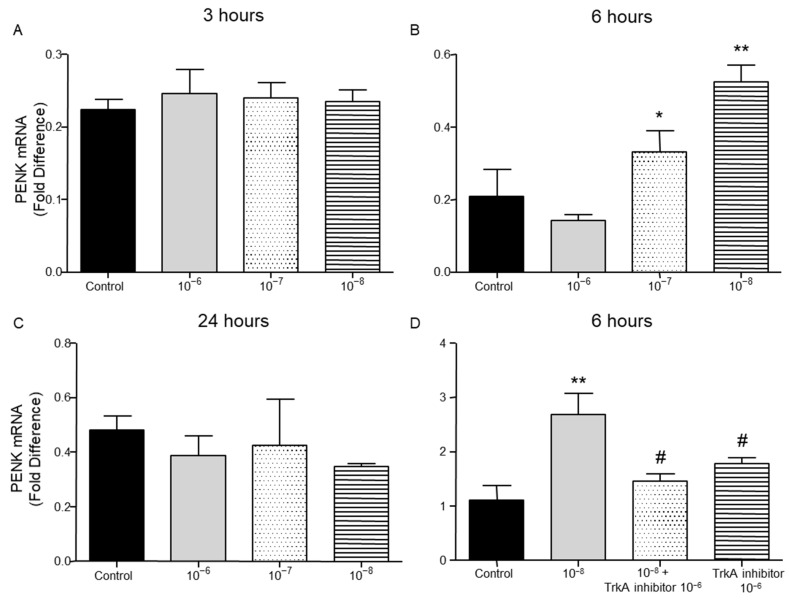
Effect of BNN27 on PENK. Administration of BNN27 10^−7^ M and 10^−8^ M increased PENK mRNA at 6 h (**B**), whereas at 3 and 24 h BNN27 did not alter its levels (**A**,**C**). Inhibition of TrkA resulted in decreased PENK mRNA levels at 6 h compared to BNN27-treated group (**D**). (*) Represents comparison between control group and BNN27-treated group, (#) represents comparison between BNN27-treated group and TrkA inhibitor, and BNN27 + TrkA inhibitor-treated groups. (**A**) In all groups n = 3; (**B**) in all groups n = 3; (**C**) in all groups n = 3; (**D**) control group n = 4, BNN27 10-8 M n = 4, BNN27 + TrkA inhibitor n = 3, TrkA inhibitor n = 4. In all panels: * *p* < 0.05, ** *p* < 0.01. ^(#)^ *p* < 0.05. Statistical analysis was performed using 1-way ANOVA and Newman–Keuls post-hoc test. Data are expressed as mean ± SEM.

PENK and POMC mRNA levels were found significantly increased following the addition of BNN27 to T lymphocytes culture. Specifically, at 3 h POMC mRNA levels were significantly elevated following treatment with 10^−8^ M BNN27 compared to control group (Figure 8A), whereas PENK mRNA levels were unchanged (Figure 9A). At 6 h, an addition of BNN27 10^−7^ M resulted in increased synthesis of both POMC (Figure 8B) and PENK (Figure 9B) mRNA levels. At the same time point, treatment with BNN27 10^−8^ M increased PENK mRNA levels (Figure 9B). Twenty-four (24) hours following treatment of T lymphocytes with BNN27, no changes in opioid peptide levels were observed at any of the concentrations studied (Figure 8C and Figure 9C). The addition of BNN27 10^−8^ M simultaneously with the TrkA receptor inhibitor significantly reduced the mRNA levels of both POMC (Figure 8D) and PENK (Figure 9D) 6 h after the treatment.

### 2.5. Effect of BNN27 on AKT Pathway

**Figure 10:**
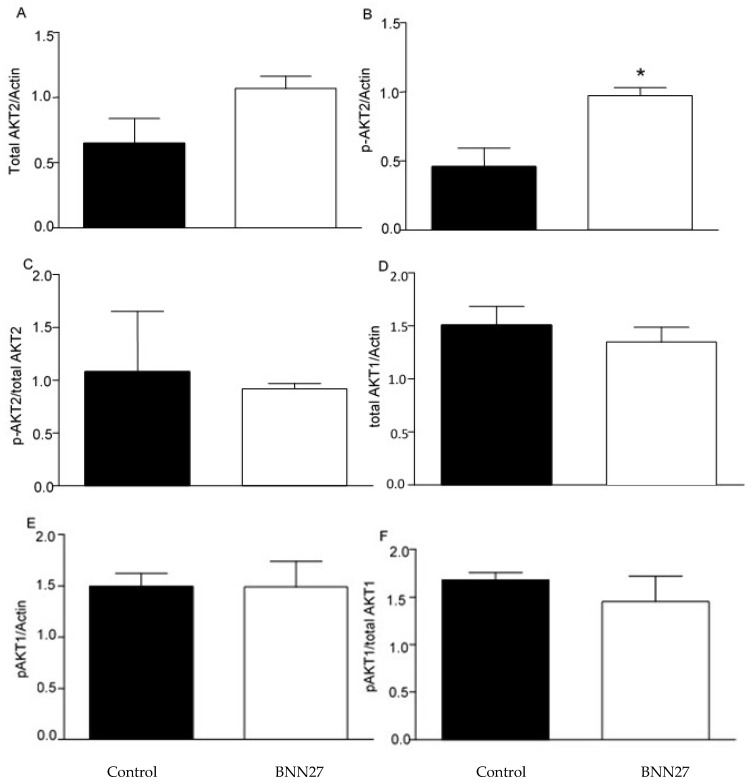
Effect of BNN27 on AKT. Treatment with BNN27 10^−8^ M increased the phosphorylated form of AKT2 (**B**), while total AKT2 (**A**) as well as AKT1 total (**D**) and phosphorylated (**E**) showed no changes in their protein levels. (**A**) In all groups n = 3; (**B**) in all groups n = 3; (**C**) in all groups n = 3; (**D**) in all groups n = 3; (**E**) in all groups n = 3; (**C**) Ratio of phosphorylated AKT2 to total AKT2; (**F**) Ratio of phosphorylated AKT1 to total AKT1; in all groups n = 3. (*) Indicates a statistically significant difference between BNN27-treated group (open bars) and control group (black bars). In all panels * *p* < 0.05. Statistical analysis was performed using unpaired t-test. Data are expressed as mean ± SEM.

Previous studies have shown that BNN27 affects the AKT signalling pathway [20,21]. Therefore, we studied the effect of BNN27 on AKT1 and AKT2 on cultured T lymphocytes. Our results showed that BNN27 at the concentration of 10^−8^ M did not affect the expression of total and phosphorylated AKT1 6 h after its addition to T lymphocytes culture; however, it increased the phosphorylated form of AKT2 at the same concentration and time point (Figure 10).

## 3. Discussion

In the present study we aimed to evaluate the effect of the synthetic analogue of DHEA, BNN27, on T lymphocytes derived from mouse spleens following induction of local inflammation and pain. Our results show that BNN27 affected T lymphocytes proliferation in a time- and dose-dependent manner. In addition, BNN27 induced cytokines secretion and triggered opioid synthesis by T cells. Several studies support the role of DHEA on proliferation and survival of various cell types and BNN27 (analogue of DHEA) has been shown to mimic this characteristic [20,22]. Regarding lymphocytes, previous studies exploring the role of DHEA on their proliferation have yielded controversial results, since it was shown to induce both lymphocyte proliferation [14] and cell death [14,23]. In our experiments we showed that BNN27 retained the dual nature of DHEA on T lymphocytes proliferation, since in concentrations of 10^−6^ M and 10^−8^ M it decreased the proliferation of T-lymphocytes without affecting their survival, while at the concentration of 10^−7^ M it stimulated T cell proliferation 48 and 72 h following its addition in the culture.

It is well documented that DHEA acts as an immunomodulator, since several studies suggest that it exerts both positive and negative effects on cytokine secretion. In detail, pre-incubation of human or mouse T lymphocytes with DHEA before stimulation with an antigen or mitogen induced an increase in IL-2 levels [16,24]. However, in 2014, Pratchke and his team demonstrated that DHEA inhibited the secretion of IL-2 and IL-10 by T lymphocytes derived from samples of patients who had undergone abdominal surgery [16]. In addition, DHEA reduced TNF-α levels in peritoneal cells following LPS stimulation [25]. Our results regarding the effect of BNN27 on the secretion of cytokines by T lymphocytes showed that BNN27 stimulated the release of IL-6 and TNF-α, since their levels were found to be significantly elevated. In addition, in accordance with Cheng’s experiments where DHEA induced an increase in IL-10 levels in cultured splenocytes [26], BNN27 in our study stimulated IL-10 synthesis by T lymphocytes.

T lymphocytes express opioid receptors (μ, κ, δ) and their treatment with opioids such as morphine may stimulate various reactions such as induction or inhibition of cytokine secretion [27,28,29]. The main receptor contributing to these processes is μ opioid receptor, since studies have shown that the most clinically administered opioids act through μ-receptor. Additionally, studies have shown that in transgenic animals lacking μ opioid receptor, most immunomodulatory properties of morphine such as phagocytosis and secretion of TNF-α by macrophages [28] have been absent. It is interesting also that cytokines such as TNF-α and IL-4 induce μ opioid receptor expression in T and B lymphocytes [30,31]. Our results are in agreement with these studies since we demonstrated that BNN27 triggered synthesis of μ opioid receptor and synthesis of its ligands POMC and PENK. These findings are also in agreement with previous published data of our team which supported the interaction of BNN27 with the opioid system in an in vivo model of inflammatory pain [32].

The effect of BNN27 on T lymphocytes was also studied following pharmacological inhibition of TrkA receptor, since previous studies support that BNN27 exerts its actions through binding to TrkA [21,32]. Assessing the survival and proliferation rate of T lymphocytes following co-administration of BNN27 with TrkA inhibitor, we found a significantly reduced proliferation rate compared to T lymphocytes treated only with BNN27. Previous studies have shown that BNN27 induces survival in neural cells in vitro and this effect is mediated through TrkA [20,22]. Our results provide evidence for the first time that BNN27 induced proliferation of immune cells in vitro, via TrkA. In addition, concomitant administration of BNN27 and TrkA antagonist inhibited the secretion of cytokines TNF-α and IL-6, as well as the synthesis of the opioid peptides POMC, PENK and μ opioid receptor by T lymphocytes. However, it is not entirely clear whether the decrease in cytokine and opioids levels was solely due to the inhibition of BNN27 activity, since T lymphocytes treated only with TrkA inhibitor exhibited again reduced IL-6 secretion and opioid peptide synthesis compared to those treated only with BNN27.

In conclusion, the new synthetic analogue of DHEA, BNN27, enhances or inhibits proliferation of mouse T lymphocytes, dependent on the concentration of treatment. In addition, it stimulates the release of cytokines and synthesis of opioids from T lymphocytes. Its mechanism of action partially involves TrkA receptor and its downstream AKT pathway, but its mode of action needs further investigation.

## 4. Materials and Methods

### 4.1. Laboratory Animals

Experiments were carried out in adult (8–12 weeks) male mice of C57BL6x1291Sv genetic background. Mice were maintained on a 12:12 h light–dark schedule and room temperature at 22 ± 2 °C, with food and water ad libitum. Experiments and animal care had been approved by the Committee of Experimental Animal Care and Protocols of the University of Crete, Greece, the Veterinary Department of the Region of Crete, Greece, under license number 147152 (date 17 July 2017, Heraklion, Crete, Greece). Furthermore, all experiments were in accordance with the International Association for the Study of Hyperalgesia and the paper follows the rules of the Declaration of Helsinki.

### 4.2. Induction of Inflammation and Harvest of Spleen Cells

Inflammation and activation of cellular immunity was induced by intraplantar injections of 20 μL of CFA (Sigma, Taufkirchen, Germany) into the left hind paw of each mouse. Six (6) hours following CFA injections mice were euthanized by cervical dislocation and spleens were harvested in ice-cold PBS. Single cell suspensions were induced by mechanical dissociation of spleens and red blood cells were removed by repeated washes with buffer containing 0.8 g NH_4_Cl, 0.084 g NaHCO_3_, 0.037 g EDTA diluted in ddH_2_O in a final volume of 100 mL. Subsequently, spleen cells were resuspended in RPMI medium (Biosera, Cholet, France) containing 10% FBS (Gibco, Massachusetts, MA, USA), 1% penicillin/streptomycin (Gibco, USA), 1% Sodium Pyruvate (Biosera, Cholet, France). Stimulation of T cell proliferation was induced by the addition of 4 μg/mL concanavalin-A (Sigma, Missouri, USA) in the culture media [33] and cells were placed in an incubator at 37 °C and 5% CO_2_. The following day 0.5 ng/mL IL-2 (Peprotech, Rocky Hill, NJ, USA) were added in culture media and spleen cells were let in an incubator at 37 °C and 5% CO_2_ for about a week to proliferate and differentiate into T cells [34,35]. Culture media containing IL-2 were added when necessary.

### 4.3. Treatment with BNN27 and TrkA Inhibitor

Following proliferation and differentiation, cells were resuspended in culture medium without FBS and placed into 24 well-plates (10^6^ cells/well). BNN27 was diluted in DMSO at the concentration of 10^−2^ M followed by further dilutions in culture media at the final concentrations of 10^−6^ M, 10^−7^ M and 10^−8^ M. To inhibit NGF receptor TrkA, a specific TrkA inhibitor (CAS 388626-12-8) (Merck, Taufkirchen, Germany) diluted in DMSO at the concentration of 10^−2^ M followed by dilution in culture media at the final concentration of 10^−6^ M was added. T cells were harvested at different timed points (3, 6, 12, 24 h following treatment) for further studies.

### 4.4. Quantitative Real-Time PCR, RT-PCR

Total RNA was extracted from T cells with Trizol reagent (Invitrogen, Waltham, MA, USA) and cDNAs were synthesized using a TAKARA PrimeScript 1st strand cDNA synthesis kit (Takara Bio, Saint-Germain-en-Laye, France). Expression of each gene of interest was determined using SYBR Green master mix (Kapa Biosystems, Wilmington, MA, USA) containing specific sets of primers in a final volume of 10 μL. Expression of each gene was normalized to β-actin mRNA. Amplification conditions included denaturation at 95 °C for 2 min followed by 40 cycles at 95 °C for 30 s and at 60 °C for 30 s. The sequence of the primers used is listed in Table 1.

### 4.5. Measurement of Cytokines

T cells were centrifuged for 5 min at 4 °C and 3000 rpm and the supernatant was collected. Bradford assay was performed to determine the total protein concentration of each sample, and quantitation of tissue TNF-α and IL-6 levels was carried out using mouse ELISA kits (Biolegend, San Diego, CA, USA) according to the instructions of the manufacturer.

### 4.6. MTT and Trypan Blue Assays

T cells were plated in 96-well plates at a concentration of 250.000 cells/well. Following incubation of cells with BNN27 and TrkA inhibitor for 24–96 h, MTT (3-(4,5-dimethylthiazol-2-yl)-2,5-diphenyltetrazolium bromide) was added at a final concentration of 0.5 mg/mL and the cells were incubated for 4 h at 37 °C. Crystals formed due to metabolism of MTT by mitochondria of living cells were dissolved by incubating the cells with DMSO at room temperature for 20 min and determining the optical density at 595 nm. Cell viability was evaluated with Trypan Blue assays. Briefly, following incubation with vehicle or BNN27, the cells were centrifuged and resuspended in culture media. An amount of the cell suspension was mixed with 0.4% solution of Trypan Blue; a small aliquot of the mixture was loaded on a hemocytometer and was examined under a microscope.

### 4.7. Western Blot

T cells were centrifuged for 5 min at 4 °C and 3.000 rpm and the cell pellet was lysed by incubation in RIPA buffer (0.1% SDS, 1% Igepal CA-630, 1% sodium deoxycholate, 10 mM Tris-HCl, pH 7.5, 150 mM NaCl, 2 μg/mL aprotinin, 1 μg/mL leupeptin, 100 μg/mL PMSF, 0.5 mM EDTA) and subsequently centrifuged for 10 min at 4 °C and 12.000 rpm. Protein concentration was determined by Bradford assay, and 30 μg of protein from each sample was loaded in 10% SDS-page gel. Proteins were transferred to a nitrocellulose membrane and blocked with 5% BSA for 1 h at 4 °C. Following washes in TBS-T, membranes were incubated overnight at 4 °C with antibodies for AKT1 and AKT2 (total/phospho 1:1000, rabbit; Cell Signaling, Danvers, MA, cat# 2964S and 8599S, respectively). To detect the bands of interest, we used the Benchmark Pre-Stained Protein Standard (Invitrogen, Waltham, MA, USA, cat # 10748-010). Normalization was carried out with β-actin (1:5000, mouse; Abcam, Cambridge, United Kingdom, cat# ab6276). Quantification of gels was performed using the Image-J software (https://imagej.net/ij/). The bands that are presented in the results proceeded from cropping and merging bands from the same original images.

### 4.8. Statistical Analysis

All data were expressed as mean ± SEM. Experiments were performed independently at least three times, and each experiment included at least n = 3 per group. Proliferation rate was analyzed using 2-way ANOVA followed by post-hoc tests. One-way ANOVA was used in all other comparisons. A *p*-value of less than 0.05 was assumed to be significant.

## Figures and Tables

**Table 1:** Sequences of RT-PCR primers.

GENE	FORWARD	REVERSE
*beta-actin*	tctctttgatgtcacgcacg	tcagaaggactcctatgtgg
*pomc*	gctgcttcagacctccatagatgtg	cagcgagaggtcgagtttgc
*penk*	cgacatcaatttcctggcgt	agatccttgcaggtctccca
*μ-opioid receptor*	acgctcagacgttccattct	tccaaagaggcccactacac
*ngf*	cacccacccagtcttcc	ctcggcacttggtctcaaa
*trka*	cctgcaacgcttggagtttg	cactcttcacgatggttaggct
*Il10*	gcgctgtcatcgatttctcccctg	ggccttgtagacaccttggtcttgg

## Data Availability

The datasets used and/or analysed during the current study are available from the corresponding author on reasonable request.
